# Dr. Charles W. Hayt

**Published:** 1901-09

**Authors:** 


Dr. Charles W. Hayt, of Corning, N. Y., was lost from a steamboat of the Cleveland and Buffalo line, during the night of

July 23-24, 1901, and has not been heard from. He graduated at the College of Physicians and Surgeons, New York, in 1899, and was a young man of great professional promise.

